# Assessment of the axilla in primary operable breast carcinoma

**DOI:** 10.1186/bcr2999

**Published:** 2011-11-04

**Authors:** M Twomey, S Hayes, TJ Browne, L Duddy, P Smiddy

**Affiliations:** 1 Cork University Hospital, Wilton, Cork, Ireland

## Introduction

The purpose of this study was to analyse assessment of the axilla in patients with primary operable breast cancer in a one-stop symptomatic breast unit.

## Methods

A retrospective review of 229 patients diagnosed with new primary operable breast carcinoma over a 12-month period. All patients underwent axillary ultrasound (US). All cases with normal US had subsequent sentinel lymph node biopsy (SLNB). All cases with abnormal US underwent fine needle aspiration cytology (FNAC). Findings were correlated with SLNB and axillary node clearance histology.

## Results

A total of 128 patients had normal US and subsequent SLNB, this was positive in 29 (23%); 12 (40%) had micro metastases; primary tumour size was >2 cm in 22 (76%). Positive predictive value for US and FNAC was 97%. Negative predictive value was 75%. False positive and negative rates for US axilla are discussed with analysis of tumour subgroups and pattern of positive nodes.

## Conclusion

The combination of US and FNAC is a powerful predictive tool of axillary disease [1]. Disappointingly false negative axillary US did not correlate solely with micro metastases. This may reflect a learning curve effect, and will be reassessed on follow-up data.
